# Supplementary material for: Identification of sources of male sterility in the Colombian Coffee Collection for the genetic improvement of Coffea arabica L
Source: PLoS One. 2023 Sep 8;18(9):e0291264. doi: 10.1371/journal.pone.0291264 (PMC10490922; doi:10.1371/journal.pone.0291264)
Supplement: Attachment — Submitted filename: Comments PONE-D-23-12131.doc [file pone.0291264.s003.doc]

**Comments on Article No. PONE-D-23-12131**

**Field preselection**

**Reference Line No 87-89**

How the F_2_BC_2_ and F_6_BC_1_ were derived should be made more clear. Which species was used as male parent for generating Back cross generations?

**Reference Line No 94-97**

**For carrying out staining and detailed observation**

Of the preselected genotypes how many floral buds/ flowers per genotype were sampled

for observation in greater detail under a microscope.

**In line no 100 &101**

For observation in greater detail under a microscope procedure was repeated at least three times for two or three flowering periods. Here flowering period should be defined.

**Controlled pollination**

**Line No** **108:**

How directed crosses (DC - fertile x male-sterile) were generated using male sterile parent as male parent ?

**Line No** **116:**

In the DC (DC - fertile x male-sterile) male parent used is “male sterile” so there is no question of fruit formation ! Please clearify.

**Examination of female fertility**

**Line No. 135:**

How male sterile genotype was used as male parent iin case of reciprocal crosses

**Staining and detailed observation**

**Line** No 170-172:

**Absence of pollen**

In the study, genotype from interspecific hybridization had small flowers, irregular leaves, absence of pollen and almost total absence of fruits should not have been discarded. These should have been pollinated with the pollen from male fertile genotypes in order to identify the male sterile or maintainer genotype.

**Line No 322 to 332:**

More clarity of expression is required.

**Line No 341 to 343 :**

Meaning of the sentence is not clear.

**Line No 363 to 365:**

Meaning of the sentence is not clear.

**Whether following was to be stated !**

Crosses between *C. arabica* (4x = 44) and other diploid species of the genus (2x = 22) leads to formation of triploids (3x = 33) that occasionally produce fruits, but after one or two back crosses with *C. arabica*, ploidy and fertility are recovered [37].

**Recommendation:**

The article needs to be improved with respect to clarity of expression. The article can be reduced further by removing the generalized statements which are not related to the aspects studied in this study. Particularly in the discussion part for example results of studies on the inheritance of male sterility have been given which is not being studied in this study. Thus, Discussion part should be reduced by removing the detailed information on inheritance aspect. The focus of the Results and Discussion should be only on the analysis of the results obtained for identification of new male sterile genotypes and their characteristic features and how they can be utilized further.

Suggested corrections may be incorporated, comments may be responded and article may be improved and resubmitted.
