# Supplementary material for: Identification of sources of male sterility in the Colombian Coffee Collection for the genetic improvement of Coffea arabica L
Source: PLoS One. 2023 Sep 8;18(9):e0291264. doi: 10.1371/journal.pone.0291264 (PMC10490922; doi:10.1371/journal.pone.0291264)
Supplement: Attachment — Submitted filename: Response to Reviewers.docx [file pone.0291264.s004.docx]

August 3, 2023

Manizales, Colombia

Dear

Abhishek Bohra
Academic Editor
PLOS ONE

We appreciate the interest shown in our manuscript and the opportunity given to us to resubmit it with major revisions.

With this communication, we are providing a point-by-point response to the comments made by the editor and academic reviewers on the article titled 'Identification of sources of male sterility in the Colombian Coffee Collection for the genetic improvement of *Coffea arabica* L.'. We are grateful for the valuable contributions made by the academic reviewers, which undoubtedly enriched the manuscript.

We hope that the changes made will allow the manuscript to meet the publication criteria of the journal PLOS ONE.

**Response to editor comments:**

Journal Requirements:

When submitting your revision, we need you to address these additional requirements.

1. Please ensure that your manuscript meets PLOS ONE's style requirements, including those for file naming. The PLOS ONE style templates can be found at

[https://journals.plos.org/plosone/s/file?id=wjVg/PLOSOne_formatting_sample_main_body.pdf](https://journals.plos.org/plosone/s/file?id=wjVg/PLOSOne_formatting_sample_main_body.pdf) and

[https://journals.plos.org/plosone/s/file?id=ba62/PLOSOne_formatting_sample_title_authors_affiliations.pdf](https://journals.plos.org/plosone/s/file?id=ba62/PLOSOne_formatting_sample_title_authors_affiliations.pdf)

Response: The manuscript complies with the requirements of the journal.

2. In your Methods section, please provide additional information regarding the permits you obtained for the work. Please ensure you have included the full name of the authority that approved the field site access and, if no permits were required, a brief statement explaining why.

Response: A brief statement regarding the access requirements provided by the Research Center has been included in the manuscript.

3. Thank you for stating the following financial disclosure:

“This study was supported by The National Coffee Research Center (Cenicafé) (Crossref Funder ID 100019597)”

Please state what role the funders took in the study.  If the funders had no role, please state: "The funders had no role in study design, data collection and analysis, decision to publish, or preparation of the manuscript."

If this statement is not correct you must amend it as needed.

Please include this amended Role of Funder statement in your cover letter; we will change the online submission form on your behalf.

Response: The role of the funding agency is included in the cover letter

4. We note that you have stated that you will provide repository information for your data at acceptance. Should your manuscript be accepted for publication, we will hold it until you provide the relevant accession numbers or DOIs necessary to access your data. If you wish to make changes to your Data Availability statement, please describe these changes in your cover letter and we will update your Data Availability statement to reflect the information you provide.

Response: The DOIs will be included

5. Please remove your figures from within your manuscript file, leaving only the individual TIFF/EPS image files, uploaded separately. These will be automatically included in the reviewers’ PDF.

Response: Figures removed within the manuscript.

6. Please include captions for your Supporting Information files at the end of your manuscript, and update any in-text citations to match accordingly. Please see our Supporting Information guidelines for more information: [http://journals.plos.org/plosone/s/supporting-information](http://journals.plos.org/plosone/s/supporting-information).

Response: Captions for supporting information were included according to the journal's format.

Additional Editor Comments:

Experts have provided their feedback on the manuscript. I encourage authors to submit a revision based on the suggestions offered by the two Reviewers. Please find below the Reviewer's comments:

Reviewer comments #1:

**Field preselection**

**Reference Line No 87-89**

How the F_2_BC_2_ and F_6_BC_1_ were derived should be made more clear. Which species was used as male parent for generating Back cross generations?

Response: A clarification is made in the manuscript.

**Reference Line No 94-97**

**For carrying out staining and detailed observation**

Of the preselected genotypes how many floral buds/ flowers per genotype were sampled

for observation in greater detail under a microscope.

Response: A clarification is made in the manuscript.

**In line no 100 &101**

For observation in greater detail under a microscope procedure was repeated at least three times for two or three flowering periods. Here flowering period should be defined.

Response: the term "flowering period" is mentioned three times, and its definition is provided in the first occurrence.

**Controlled pollination**

**Line No** **108:**

How directed crosses (DC - fertile x male-sterile) were generated using male sterile parent as male parent?

Response: A clarification is made in the manuscript.

**Line No** **116:**

In the DC (DC - fertile x male-sterile) male parent used is “male sterile” so there is no question of fruit formation ! Please clearify.

Response: When well-defined male-sterile plants are used, the outcome in the DC (DC - fertile x male-sterile) may seem obvious, as well as in the case of self-fertilization. However, the male-sterile condition in the preselected genotypes had not been confirmed, so it was possible for fruits to form, as observed in ten out of the 23 genotypes used.

A clarification is made in the manuscript.

**Examination of female fertility**

**Line No. 135:**

How male sterile genotype was used as male parent iin case of reciprocal crosses.

Response: Given the defined male-sterile condition, in the reciprocal crosses, it acted as the pollen receptor. Despite the natural absence of pollen, emasculation was performed to equalize the treatment with the genotype used as a control, so that any negative effect caused by this practice would affect both genotypes equally.

**Staining and detailed observation**

**Line** No 170-172:

**Absence of pollen**

In the study, genotype from interspecific hybridization had small flowers, irregular leaves, absence of pollen and almost total absence of fruits should not have been discarded. These should have been pollinated with the pollen from male fertile genotypes in order to identify the male sterile or maintainer genotype.

Response: The characteristics of the mentioned plant indicated that beyond male-sterile, the absence of pollen was caused by total sterility of the plant, possibly due to chromosomal aberrations given its hybrid origin. In addition, this genotype, like the other plants used in this study, was surrounded by fertile plants, so if it were male-sterile, the absence of fruits would not have been highly compromised. No modification is made to the manuscript.

**Line No 322 to 332:**

More clarity of expression is required.

Response: The sentence is reframed in the revised manuscript. A bibliographic reference is updated in this section.

Modified: Centro Nacional de Investigaciones de Café Informe Anual Cenicafé 2021 Colombia: El Centro; 2021, by: Gómez JH, Benavides P, Maldonado JD, Jaramillo J, Acevedo FE, Gil ZN. Flower-Visiting insects ensure coffee yield and quality. Agriculture. 2023;13: 1392. doi:10.3390/agriculture13071392

**Line No 341 to 343 :**

Meaning of the sentence is not clear.

Response: A sentence is removed, and a paragraph is relocated to enhance the clarity of the discussion.

**Line No 363 to 365:**

Meaning of the sentence is not clear.

**Whether following was to be stated !**

Crosses between *C. arabica* (4x = 44) and other diploid species of the genus (2x = 22) leads to formation of triploids (3x = 33) that occasionally produce fruits, but after one or two back crosses with *C. arabica*, ploidy and fertility are recovered [37].

Response: It is adjusted in the manuscript to improve the clarity of the sentence.

**Recommendation:**

The article needs to be improved with respect to clarity of expression. The article can be reduced further by removing the generalized statements which are not related to the aspects studied in this study. Particularly in the discussion part for example results of studies on the inheritance of male sterility have been given which is not being studied in this study. Thus, Discussion part should be reduced by removing the detailed information on inheritance aspect. The focus of the Results and Discussion should be only on the analysis of the results obtained for identification of new male sterile genotypes and their characteristic features and how they can be utilized further.

Suggested corrections may be incorporated, comments may be responded and article may be improved and resubmitted.

Response

We sincerely appreciate the careful attention given to the review of our manuscript; the valuable suggestions provided have undeniably contributed to enhancing its clarity, particularly for readers who may not be familiar with the species. As a result of the feedback received, irrelevant sections of the discussion were omitted, and the manuscript was further strengthened by emphasizing the significance of the obtained results. Additionally, we have now incorporated a comprehensive analysis of the advantages and limitations related to the utilization of male-sterile and partially male-sterile genotypes in our study.

Revisor #2:

General observations:

(i) This paper reports the finding of a male sterility system, arising naturally and through wide hybridizations. Besides, some partial male sterile plants were also found. Various morphological details related to flower and anther types are described well. The authors also suggest its potential use in the genetic enhancement of coffee yields. Since this is the first such discovery, it recommend its publication. (ii) At more than one places there are repetitions of subject and text lines. Authors need to consider editing the text.

Scientific observations.

The authors should make clear in the abstract, objective, introduction that this paper deals with a genetic male sterility (GMS). It is better that the text portions be slightly modified accordingly.
At present the discussion portion appears quite weak. It should be strengthened by incorporating information on the issue of its potential utilization in crop improvement, particularly in view of its autogamous breeding nature. Although the authors have provided information on this subject using some passing references, but, I feel, the paper will be complete in itself if brief information on the large-scale seed production the male steriles / hybrids and their limitations in coffee are included. Besides information on the reported heterosis for yield will add to the overall quality
of the paper. And finally, is there any adverse effect on the quality of beans?

Recommendation
This manuscript may be published after the revision.

Response

Thank you for the time taken to review our manuscript and for the important observations provided. Below, we are presenting our response to the various suggestions:

For the sections that we considered repetitive, we unified them into a single paragraph within the discussion. Similarly, in some cases, sentences were relocated to improve clarity and enhance coherence between sections.

While it is suggested to specify in different sections of the manuscript that we are dealing with genetic male-sterility (GMS), the suggestion is only included in the part of the discussion where we mention, based on our observations, that fertility is reiterated in the F1 generation. In general, further investigation into this aspect is underway, as it is the first time that a considerable number of sources have been available to do so.

The suggestion to strengthen the discussion according to the mentioned points effectively improved the quality of the manuscript, and we appreciate this valuable input.

Regarding your inquiry about the potential negative impact of androsterility sources on beverage quality, it holds great significance, given the natural evolution of the market. This aspect corresponds to a second stage of our study, as well as other points you have pointed out. It is worth noting that existing experiences with the inclusion of Ethiopian germplasm have shown favorable results, and in no case have they indicated any negative effect on beverage quality.
